# Major ozone therapy improves disease severity without affecting retinal microvascular parameters in fibromyalgia syndrome: an observational pre-post study

**DOI:** 10.1007/s00296-026-06145-w

**Published:** 2026-05-23

**Authors:** Burhan Fatih Kocyigit, Gülşah Yaşa Öztürk, Duygu Topaktaş Emekli, Eda Sahutoglu

**Affiliations:** 1 Department of Physical Medicine and Rehabilitation, University of Health Sciences, Adana City Research and Training Hospital, Adana, Turkey; 2 Department of Ophthalmology, Adana City Training and Research Hospital, Adana, Turkey; 3 Department of Ophthalmology, Osmaniye Training and Research Hospital, Osmaniye, Turkey

**Keywords:** Fibromyalgia, Ozone, Complementary therapies, Ophthalmology, Optical coherence tomography, Retinal vessels, Microcirculation

## Abstract

This study aimed to assess the impact of ten sessions of major ozone therapy on disease severity and ophthalmologic parameters in patients with fibromyalgia syndrome (FMS). This observational study was conducted in the Physical Medicine and Rehabilitation and Ophthalmology clinics of a tertiary healthcare facility. The study involved 30 patients diagnosed with FMS who underwent 10 weekly sessions of major ozone therapy. Symptom severity was measured before and after treatment using the Fibromyalgia Impact Scale (FIQ). The study assessed ophthalmic parameters, including visual acuity, spherical equivalent, astigmatism, axis, intraocular pressure, and central corneal thickness. Additionally, microvascular changes were examined using optical coherence tomography angiography (OCTA). Post-treatment, FIQ scores decreased significantly (before: 67.19 ± 14.02; after: 46.78 ± 11.46; *p* < 0.001), indicating a notable reduction in symptom burden. The mean intraocular pressure was measured at 15.25 ± 2.67 mmHg before treatment and 15.41 ± 3.55 mmHg after treatment, while central corneal thickness was recorded as 530.50 ± 19.35 μm and 531.50 ± 19.21 μm. Superficial capillary plexus foveal vascular density was found to be 14.98 ± 7.16% before treatment and 15.34 ± 7.62% after treatment. Best-corrected visual acuity remained 1.0 ± 0.0 in both assessments. Spherical equivalent was − 0.05 ± 1.05 D pre-treatment and − 0.01 ± 1.03 D post-treatment (*p* = 0.380), and astigmatism was − 0.64 ± 0.60 D and − 0.64 ± 0.54 D (*p* = 0.100). Foveal avascular zone area (0.333 ± 0.129 vs. 0.331 ± 0.133 mm²; *p* = 0.732), total foveal retinal thickness (213 vs. 214 μm; *p* = 0.253), and deep capillary plexus foveal vascular density (30.07 ± 8.44% vs. 30.18 ± 8.18%; *p* = 0.786) also remained stable. No statistically significant differences were observed in anterior segment parameters, intraocular pressure, central corneal thickness, or retinal microvascular structures as measured by OCTA (*p* > 0.05). Ten sessions of major ozone therapy effectively reduced symptom burden in FMS patients, though short-term changes in the anterior segment and retinal microvascular structures were not measurable. Ozone therapy may serve as a complementary and supportive approach alongside existing treatments for managing FMS.

**Supplementary Information:**

The online version contains supplementary material available at 10.1007/s00296-026-06145-w.

## Introduction

Fibromyalgia syndrome (FMS) is a long-term disorder marked by widespread musculoskeletal pain, joint stiffness, fatigue, sleep issues, cognitive difficulties, and mood fluctuations. Patients often face challenges in daily activities, and their responses to treatment can vary [1]. The etiology of FMS remains incompletely elucidated; nevertheless, several factors are posited to contribute, including central vulnerability, genetic predisposition, hormonal dysregulation, and hypothalamic-pituitary-adrenal axis dysfunction. Furthermore, it has been documented that FMS patients experience disturbances in the sympathetic-parasympathetic balance, autonomic dysfunction, and subsequent systemic effects like vascular changes and blood pressure fluctuations [2, 3].

In FMS, complementary therapies can be preferred in clinical management [4]. Ozone therapy is one such approach. Ozone therapy is known for its anti-inflammatory properties and its ability to regulate oxidative stress; when administered systemically, it improves microcirculation by increasing tissue oxygenation and supporting energy metabolism. These qualities have potential benefits, especially in alleviating pain, reducing fatigue, and improving overall quality of life [5, 6].

Recent years have seen a growing interest in the ophthalmic involvement of FMS [7, 8, 9]. Studies showed that patients can experience a range of ophthalmology-related symptoms, commonly including dry eye, photophobia, and vision issues [10, 11]. These symptoms are considered to be linked to immune system changes and autonomic nervous system dysfunction. Additionally, FMS patients often exhibit reduced eye sensitivity, which may be associated with a chronic decline in tear production [12].

This study was designed with consideration of the potential ophthalmic implications of FMS, and the impact of ozone therapy on vascular structures was factored into the protocol formulation. FMS is associated with autonomic nervous system dysfunction, which has been shown to affect ocular vasculature, tear production, and corneal sensitivity. Retinal microvascular alterations in FMS have been demonstrated using optical coherence tomography angiography (OCTA) in previous studies, including those conducted by the present research group [7, 8, 9]. Given that major ozone therapy exerts systemic effects on microcirculation and tissue oxygenation, it is hypothesized that this intervention may induce measurable changes in retinal microvascular structures. In the study, FMS patients underwent 10 sessions of major ozone therapy. The Fibromyalgia Impact Questionnaire (FIQ) was used to assess the effects of ozone therapy on the clinical picture of FMS. In addition, the potential effects of major ozone therapy on vascular structures and microcirculation in the eye were evaluated through OCTA and other ophthalmologic measurements.

## Methods

This observational study was conducted at the Physical Medicine and Rehabilitation and Ophthalmology clinics of a tertiary healthcare facility. All patients included in the study were diagnosed with FMS in the Physical Medicine and Rehabilitation outpatient clinic. The study period was from May 2025 to January 2026. Only individuals who met the 2016 American College of Rheumatology (ACR) criteria were included in the FMS group [13]. The study participants aged 18 and older who consented to join. Exclusion criteria included conditions: dementia, glaucoma, retinal disorders, uveitis, iridocyclitis, history of steroid use, cardiovascular issues, hypertension, diabetes mellitus, inflammatory rheumatic or autoimmune diseases, malignancy, pregnancy, vasculitis, neurological disorders, previous ophthalmologic surgeries, and medications that could influence the results. All participants experienced routine laboratory testing. Those who did not meet the specified criteria were excluded. Verification of adherence to exclusion criteria was conducted through the hospital record system, and medication usage was confirmed via the Ministry of Health database. During the study period, patients were not administered any new drugs and were permitted to maintain their current therapies. Furthermore, informed consent was obtained from all participants using written consent forms.

Demographic data gathered from participants included age, sex, body mass index (BMI), disease duration, occupational status, and educational level. All clinical evaluations, OCTA measures, and other assessments were conducted by a proficient expert in physical medicine and rehabilitation and ophthalmology, adhering to established protocols. The FIQ was used to assess disease severity and its impact on quality of life. Physical medicine and rehabilitation and ophthalmology evaluations were performed by the same physicians.

### Fibromyalgia impact questionnaire

The FIQ was employed to evaluate the severity of FMS and its effects on patients’ quality of life. The FIQ is a validated and reliable instrument that assesses pain, fatigue, sleep patterns, physical function, psychological status, and the impact of FMS on daily activities. The scale ranges from 0 to 100; higher values indicate a greater negative impact of the condition on quality of life and symptom burden. Thanks to its multidimensional structure, the FIQ offers a thorough evaluation of the clinical progression of FMS and patients’ functional status. The scale’s validity and reliability in Turkish have been confirmed in previous studies [14].

### Ophthalmologic assessment

The Nidek UP-1000 ultrasonic pachymeter was used to evaluate central corneal thickness (CCT). Measurements were taken at the center of the cornea, and the reported result is the average of three consecutive readings in eyes without pharmacological pupil dilation. Refractive parameters, including spherical equivalent, astigmatism, and axis, were measured using an automated refractometer (Topcon KR-800 Auto Kerato-Refractometer, Tokyo, Japan). Best-corrected visual acuities were measured using a Snellen chart. Intraocular pressure was determined with a Goldmann applanation tonometer (Haag-Streit, USA) [9].

OCTA is a noninvasive, advanced imaging technique that allows detailed visualization of blood vessels by detecting blood flow within posterior segment vessels without the need for fluorescein dye. The process starts with identifying motion contrast, then acquiring successive B-scans to find differences between them. Finally, the device’s software processes this information to produce detailed images of the retinal vascular network and to correct motion artifacts caused by eye movements.

The system detects motion differences in the posterior segment tissues and calculates signal variations between stationary and moving structures, creating three-dimensional image cubes of the retina, choroid, and optic nerve. These cubes can be examined layer by layer using the device’s segmentation features. In this study, OCTA scans were performed by the same trained operator with the AngioVue SD-OCT device (Optovue Inc., Fremont, CA, USA), which operates at a wavelength of 840 nm, with 5 μm axial and 15 μm transverse resolutions, and a scan rate of 70 kHz. Only 3 × 3 mm images with a scan quality score of 8 or higher were included, and images with segmentation, motion, or projection artifacts were excluded.

The device’s phase evaluation tool automatically quantified the foveal avascular zone (FAZ) magnitude (mm²), FAZ perimeter (mm), and foveal density (%). Vascular density (%) was assessed utilizing the density evaluation method for the foveal and parafoveal areas, delineated by concentric circles with diameters of 1 mm and 3 mm centered on the FAZ, for both the superficial and deep capillary plexuses. Each area was subdivided into four quadrants: temporal, superior, nasal, and inferior. Blood flow in the outer retina and choriocapillaris was quantified in mm² using the flow assessment tool within a 1 mm radius, corresponding to an area of 3.142 mm² [15, 16].

**Fig. 1:**
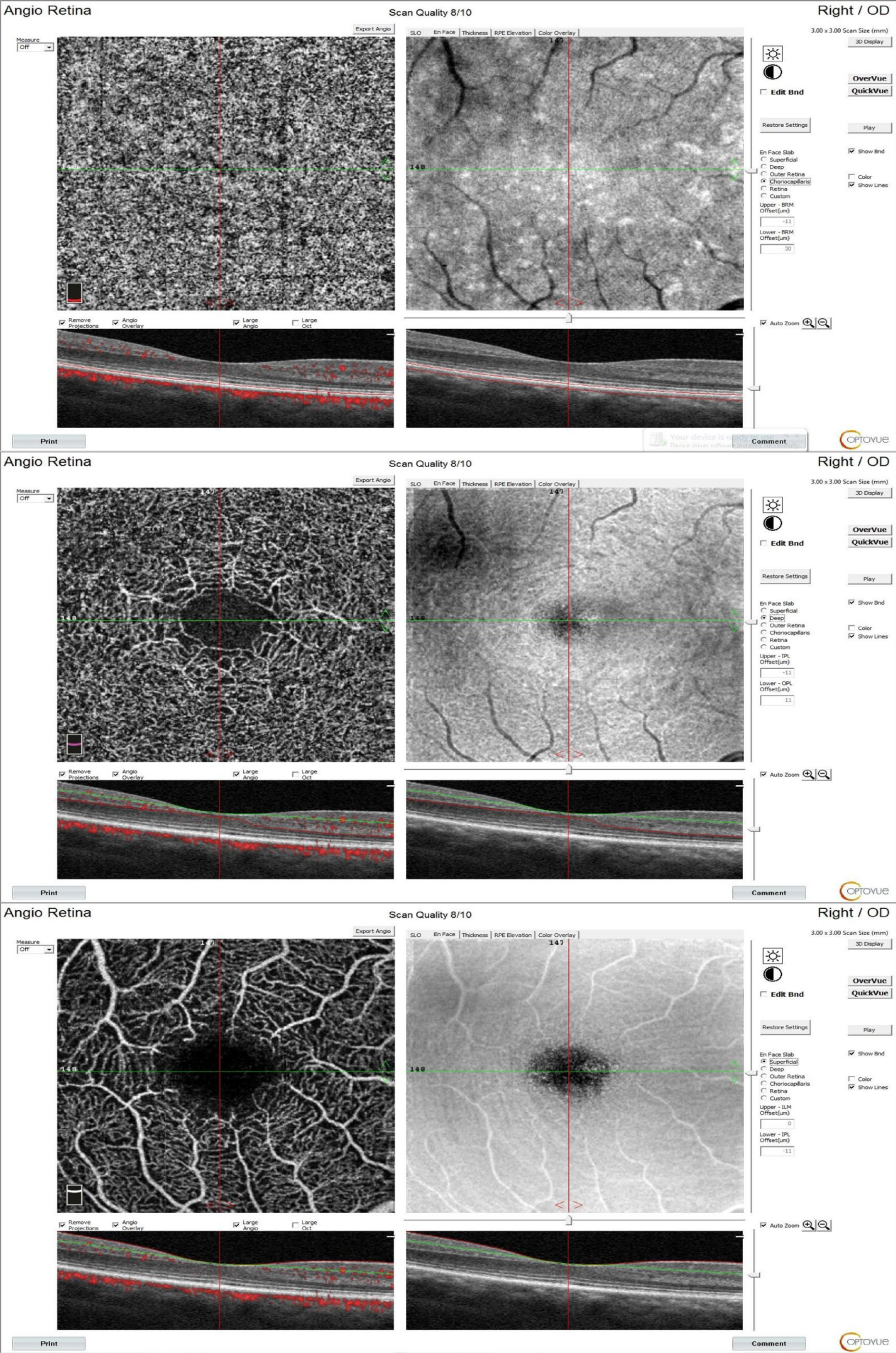
Example optical coherence tomography angiography images demonstrating retinal microvascular structures in fibromyalgia patients

Representative examples of OCTA images from the participants are displayed in Fig. 1.

### Ozone administration

Major ozone autohemotherapy procedures were carried out using the OZONOSAN Photonic ozone generator (Hansler GmbH, Germany). This compact desktop device features automatic calibration and a digital display system, capable of producing ozone in the range of 2–80 µg/mL. Medical ozone is generated by converting diatomic oxygen (O₂) into triatomic ozone (O₃) through an electrical discharge in the ozone generator, utilizing oxygen supplied from a medical oxygen cylinder.

In each session, venous access was established using a 21-G ozone-resistant butterfly needle. About 100 mL of venous blood was drawn into a vacuum glass bottle containing sodium citrate to prevent clotting. After blood collection, ozone gas at 20 µg/mL - sourced from an ozone generator - was added to the bottle using a transfer set fitted with a bacterial filter and a siliconized ozone-resistant syringe. The ozone was evenly distributed in the blood through a microbubble system, creating a large reactive surface. The blood in contact with ozone was ozonized in a closed, sterile environment and then reinfused into the patient via the same venous route, using a specialized blood transfusion set, within approximately 10–15 min.

At our Traditional and Complementary Medicine Application Center, ozone therapy was administered to patients with FMS at a concentration of 20 µg/mL, following the dosage protocol recommended by the German “Medical Society for the Use of Ozone in Prevention and Therapy.” The treatment involved 10 sessions, once a week [17, 18].

**Table 1:** The baseline features of the fibromyalgia group

Features	*n* (%) or Mean ± SD	Median (Minimum-Maximum)
Age (years)	52.8 ± 7.8	53 (31–70)
BMI (kg/m²)	26.8 ± 3.2	25.7 (22.1–37.1)
Sex (n) (%)		
Female	28 (93.3)	
Male	2 (6.7)	
Symptom duration (years)	9.3 ± 4.2	8 (4–19)
Educational status (n) (%)		
Primary School	9 (30)	
Middle School	4 (13.3)	
High School	15 (50)	
University or higher	2 (6.7)	
Occupational status (n) (%)		
Working	4 (14.8)	
Not working/Housewife	20 (74.1)	
Retired	3 (11.1)	

*BMI* body mass index, *kg* kilogram, *m²* square meter, *n* number, *%* percentage, *SD* standard deviation

**Table 2:** Comparison of spherical equivalent, astigmatism, axis, intraocular pressure, and central corneal thickness before and after ozone therapy in fibromyalgia patients

Parameter	Pre-ozone	Post-ozone	*p*
Best-corrected visual acuity right (decimals)	1 ± 0	1 ± 0	-
Spherical equivalent-right (diopter)	-0.05 ± 1.05	-0.01 ± 1.03	0.380
Astigmatism-right (diopter)	-0.64 ± 0.60	-0.64 ± 0.54	0.100
Axis-right (degree)	104 ± 56.21	105.38 ± 53.72	0.449
Intraocular pressure-right (mmHg)	15.25 ± 2.67	15.41 ± 3.55	0.608
Central corneal thickness-right (µm)	530.50 ± 19.35	531.50 ± 19.21	0.615

**Table 3:** Comparison of pre- and post-ozone optical coherence tomography angiography parameters in fibromyalgia patients

Parameters	Pre-ozone	Post-ozone	*p*
Total retinal foveal thickness^a^ (µm)	213 (112–271)	214 (112–272)	0.253
Total retinal parafoveal thickness^a^ (µm)	279 (217–305)	278 (200–305)	0.987
Foveal avascular zone area^*^ (mm²)	0.333 ± 0.129	0.331 ± 0.133	0.732
Foveal avascular zone perimeter ^*^(mm)	2.27 ± 0.54	2.29 ± 0.54	0.295
Foveal density^a^ (%)	50.45 (37.10–57.73)	50.31 (37.19–57.64)	0.393
SCP foveal vascular density*(%)	14.98 ± 7.16	15.34 ± 7.62	0.411
SCP parafoveal vascular density^a^ (%)	50.65 (40.4–54.2)	50.10 (37.5–53.7)	0.776
SCP parafoveal temporal vascular density^a^ (%)	48.60 (35–53.9)	48.65 (34.6–52.7)	0.517
SCP parafoveal superior vascular density^a^ (%)	52.95 (42.5–56.2)	52.75 (42.1–56.7)	0.624
SCP parafoveal nasal vascular density^a^ (%)	48.78 ± 2.84	47.90 ± 4.44	0.115
SCP parafoveal inferior vascular density^a^ (%)	52.10 (28.4–60.7)	50.80 (36.8–55.4)	0.569
DCP foveal vascular density^*^ (%)	30.07 ± 8.44	30.18 ± 8.18	0.786
DCP parafoveal vascular density^*^ (%)	55.03 ± 4.17	54.46 ± 4.05	0.326
DCP parafoveal temporal vascular density^a^ (%)	56.10 (40.2–62)	55.15 (40.1–60.8)	0.435
DCP parafoveal superior vascular density^*^ (%)	55.44 ± 3.91	54.89 ± 4.05	0.452
DCP parafoveal nasal vascular density^*^ (%)	56.1 ± 4.01	54.59 ± 4.32	0.152
DCP parafoveal inferior vascular density^a^ (%)	56.15 (41.2–61.9)	55.75 (41.1–61.1)	0.900
Choriocapillaris flow area^a^ (mm²)	2.12 (1.34–2.27)	2.12 (1.31–2.36)	0.873
Outer retinal flow area^a^ (mm²)	0.26 (0.12–1.61)	0.28 (0.12–1.64)	0.504

*SCP* superficial capillary plexus, *DPC* deep capillary plexus, *FMS* fibromyalgia syndrome

^a^Data are expressed as median (minimum - maximum)

*Data are expressed as mean ± standard deviation

### Ethical approval

The study was approved by the Traditional and Complementary Medicine Ethics Committee on April 14, 2025, under decision number 90. The study was conducted in accordance with the principles of the Declaration of Helsinki. Written informed consent was obtained from all participants prior to enrollment.

### Statistical analysis

Data analysis was conducted using SPSS version 20.0 (IBM Corp., Armonk, NY, USA). Data are presented as numbers (n), percentages (%), means ± standard deviations, and medians (minimum - maximum). The Shapiro-Wilk test was employed to evaluate the normality of the data. For comparisons of continuous variables pre- and post-ozone administration, the paired t-test was utilized for normally distributed data, whereas the Wilcoxon signed-rank test was employed for non-normally distributed data. A p-value less than 0.05 was considered statistically significant.

## Results

The study enrolled 30 patients diagnosed with FMS, of whom 28 (93.3%) were female, and 2 (6.7%) were male. The participants had a mean age of 52.8 ± 7.8 years (range, 31 to 70 years) and an average disease duration of 9.3 ± 4.2 years (range, 4 to 19 years). The demographic and clinical baseline characteristics of the cohort are presented in Table 1.

The mean FIQ score before ozone sessions was 67.19 ± 14.02, which decreased to 46.78 ± 11.46 following therapy. Paired t-test analysis revealed a statistically significant reduction in FIQ scores after ozone therapy (t = 10.13, *p* < 0.001).

Ophthalmologic measures, encompassing best-corrected visual acuity, spherical equivalent, astigmatism, axis, intraocular pressure, and CCT, were assessed before and following ozone administration. No statistically significant changes were detected between pre- and post-treatment data. The mean best-corrected visual acuity was 1.0 ± 0.0 in both measurements. Spherical equivalent was − 0.05 ± 1.05 D pre-treatment and − 0.01 ± 1.03 D post-treatment (*p* = 0.380). Astigmatism values were − 0.64 ± 0.60 D and − 0.64 ± 0.54 D (*p* = 0.100), and axis was 104 ± 56.21° and 105.38 ± 53.72° (*p* = 0.449). Intraocular pressure was 15.25 ± 2.67 mmHg pre- and 15.41 ± 3.55 mmHg post-treatment (*p* = 0.608), while CCT was 530.50 ± 19.35 μm and 531.50 ± 19.21 μm (*p* = 0.615) (Table 2).

Retinal and vascular characteristics were evaluated using OCTA before and following ozone sessions in patients with FMS. No statistically significant alterations were detected between pre- and post-treatment measures (*p* > 0.05). The total foveal retinal thickness was 213 μm (112–271) pre-ozone and 214 μm (112–272) post-ozone (*p* = 0.253), while parafoveal thickness was 279 μm (217–305) and 278 μm (200–305), respectively (*p* = 0.987). FAZ area and perimeter data were 0.333 ± 0.129 mm² versus 0.331 ± 0.133 mm² (*p* = 0.732) and 2.27 ± 0.54 mm versus 2.29 ± 0.54 mm (*p* = 0.295), respectively. The superficial capillary plexus foveal vascular density remained stable at 14.98 ± 7.16% prior to treatment and 15.34 ± 7.62% subsequent to treatment (*p* = 0.411). Parafoveal superficial capillary plexus densities, encompassing the temporal, superior, nasal, and inferior quadrants, did not exhibit significant alterations (*p* > 0.05). Furthermore, the vascular densities of the deep capillary plexus and the flow areas of the outer retina and choriocapillaris demonstrated no statistically significant differences following therapy (*p* > 0.05) (Table 3).

## Discussion

This study assessed disease severity and ophthalmologic parameters in patients with FMS following 10 sessions of major ozone therapy. Key findings showed a notable reduction in FIQ scores. However, no statistically significant changes were observed in ophthalmologic measures, including visual acuity, refractive values, intraocular pressure, CCT, or retinal vascular structures and microcirculation measured by OCTA.

The substantial reduction in FIQ scores following administration suggests a significant improvement in disease severity attributable to ozone therapy. Since the FIQ is a comprehensive scale that includes pain, fatigue, sleep issues, physical function, psychological health, and daily activities, lower scores may indicate wide-ranging clinical benefits [19]. Previous studies reported beneficial effects of ozone therapy in the management of FMS [20, 21, 22]. This finding may be linked to the biological mechanisms behind ozone therapy. Ozone decreases oxidative stress by boosting the body’s antioxidant response and promotes energy metabolism through improved microcirculation in tissues. These effects can help alleviate chronic pain, fatigue, and enhance overall functional capacity. Evidence indicates that ozone reduces pain and fatigue symptoms by modulating inflammatory markers, supporting our results [23, 24, 25]. Additionally, the notable reduction in FIQ scores reflects positive shifts in patients’ subjective perception of their quality of life. However, it is crucial to recognize that FIQ is a subjective metric and can be influenced by differences in individual perception. Therefore, assessing the effects of ozone therapy using objective biomarkers and long-term follow-up will enhance the clinical validity of the results.

Ophthalmologic evaluations after ozone therapy showed no significant changes in visual acuity, refractive parameters, intraocular pressure, and CCT. The study was designed based on ozone’s microcirculation-enhancing and tissue oxygenation-improving effects [26], expecting potential positive changes in the anterior segment and refractive parameters. However, the measured values remained similar before and after treatment. This suggests that 10 sessions of ozone therapy did not produce measurable short-term changes in the anterior segment and refractive parameters.

No significant changes were detected in retinal and vascular features assessed by OCTA following ozone therapy. Foveal and parafoveal retinal thickness, FAZ metrics, and densities in both the superficial and deep capillary plexuses remained stable after ozone sessions. These outcomes suggest that 10 sessions of ozone therapy do not induce significant short-term morphological or perfusion alterations in the microvasculature. While ozone’s systemic effects on microcirculation and tissue oxygen transport are documented [27], this study focused on a specific dose and a 10-session protocol tailored to FMS symptoms. A different regimen might be needed for tissues like the retina, which are more stable and well-perfused. Consequently, developing protocols with varying doses and session counts targeting ophthalmic structures could be essential to detect significant changes. Although the observational design with 30 participants in this study allowed us to evaluate the effects of ozone therapy, the limited sample size may have reduced our capacity to detect small or medium-sized effect sizes.

This study has several limitations. The sample size was limited to 30 participants, which may have constrained the ability to detect small or moderate effects. The study used an observational design, and the absence of a randomized controlled trial limits causal inference from the results. The ozone therapy protocol consisted of a 20 µg/mL dose administered over 10 sessions for FMS symptoms; distinct procedures with varying dosages and session numbers may be necessary to achieve quantifiable alterations in retinal and microvascular architecture. The sensitivity of OCTA and other ophthalmic tests may be constrained in identifying minor or subclinical alterations. Furthermore, individual extra-ophthalmic manifestations of FMS, including fatigue, sleep disturbance, depression, and anxiety, were not evaluated independently. Instead, the FIQ served as a comprehensive, multidimensional instrument for assessing overall symptom burden. Domain-specific subscale analysis of the FIQ was not conducted; only the total score was recorded. This limitation restricts the interpretation of treatment effects on individual symptom dimensions, including pain, fatigue, and psychological status. Ultimately, given the potential impact of individual perceptual variations on subjective measures such as FIQ, it is essential to validate observed symptom improvements with objective biomarkers to strengthen the findings.

## Conclusion

Following 10 sessions of major ozone therapy, a notable reduction in FIQ scores was observed in FMS patients, along with a considerable enhancement in short-term symptom burden. No substantial alterations were detected in anterior segment characteristics, refraction, intraocular pressure, CCT, retinal microvascular structure, and perfusion parameters assessed by OCTA. These data corroborate the symptom-alleviating benefits of the current regimen on FMS manifestations. Ozone therapy may represent as a complementary and supportive approach alongside established treatments for the complex clinical presentation of FMS. Larger sample sizes, randomized controlled trials with extended follow-up, implementation of varied dosing and session protocols, and the incorporation of both subjective and objective biomarkers will facilitate a more thorough assessment of the effects of ozone therapy on FMS and potential retinal microvascular alterations.

## Supplementary Information

Below is the link to the electronic supplementary material.

Supplementary Material 1

## Declarations

### Open data sharing note

Raw data can be shared if requested.

### Disclaimer

The authors declare that no part of this manuscript has been copied, published, or submitted for publication elsewhere, in whole or in part.
